# Ligand-Tuned AgBiS_2_ Planar Heterojunctions Enable Efficient Ultrathin Solar Cells

**DOI:** 10.1021/acsnano.4c07621

**Published:** 2024-11-27

**Authors:** Jianian Chen, Qixuan Zhong, Elise Sirotti, Guanda Zhou, Lukas Wolz, Verena Streibel, Johannes Dittloff, Johanna Eichhorn, Yongqiang Ji, Lichen Zhao, Rui Zhu, Ian D. Sharp

**Affiliations:** † Walter Schottky Institute, Technical University of Munich, Am Coulombwall 4, 85748 Garching, Germany; ‡ Physics Department, TUM School of Natural Sciences, Technical University of Munich, Am Coulombwall 4, 85748 Garching, Germany; § State Key Laboratory for Artificial Microstructure and Mesoscopic Physics, School of Physics, Frontiers Science Center for Nano-optoelectronics and Collaborative Innovation Center of Quantum Matter, Peking University, Beijing, 100871, People’s Republic of China; ∥ Peking University Yangtze Delta Institute of Optoelectronics, Nantong, 226010, People’s Republic of China; ⊥ Collaborative Innovation Center of Extreme Optics, Shanxi University, Taiyuan, 030006, People’s Republic of China

**Keywords:** AgBiS_2_ QDs, SILE strategy, bandgap alignment, *p*−*n* junction, solar cell

## Abstract

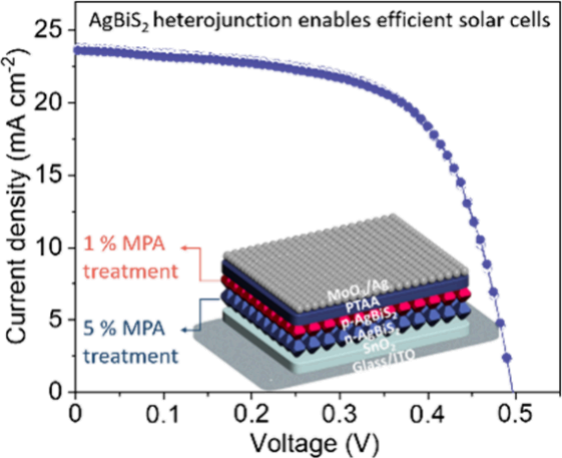

AgBiS_2_ quantum dots (ABS QDs) have emerged as highly promising candidates for photovoltaic applications due to their strong sunlight absorption, nontoxicity, and elemental availability. Nevertheless, the efficiencies of ABS solar cells currently fall far short of their thermodynamic limits due in large part to sluggish charge transport characteristics in nanocrystal-derived films. In this study, we overcome this limitation by tuning the surfaces of ABS semiconductor QDs via a solvent-induced ligand exchange (SILE) strategy and provide key insights into the role of surface composition on both *n*- and *p*-type charge transfer doping, as well as long-range charge transport. Using this approach, the electronic properties of ABS films were systematically modulated, thereby enabling the design of planar *p*–*n* heterojunctions featuring favorable band alignment for solar cell applications. Carrier transport and separation are significantly enhanced by the built-in electric fields generated within the ultrathin (30 nm) ABS heterojunction absorber layers, resulting in a notable solar-cell power conversion efficiency of 7.43%. Overall, this study presents a systematic and straightforward strategy to tune not only the surfaces of ABS, but also the electronic properties of solid-state films, thereby enabling junction engineering for the development of advanced semiconductor structures tailored for photovoltaic applications.

## Introduction

Colloidal quantum dots (CQDs) are well established as leading candidates for next-generation thin film solar cells due to their bandgap tunability and strong optical absorption that is nearly ideal for efficiently capturing solar radiation.^1,2^ However, the long insulating organic ligands that are present on the particle surfaces impede efficient charge migration within QD-derived thin films used in solar cells, leading to poor device performance.^2−4^ To address this limitation, ligand exchange methods have been widely employed to replace lengthy organic molecules on the surfaces of QDs and to tune their energetic band alignments for integration into functional devices, resulting in dramatically improved power conversion efficiencies (PCEs).^5−9^ Indeed, best-in-class PbS QDs solar cells have now achieved an impressive record PCE of 15%.^10^ Nevertheless, the inherent toxicity of lead and associated environmental concerns have motivated a vigorous quest for alternative materials.^11^

In recent years, AgBiS_2_ quantum dots (ABS QDs) have attracted growing attention for photovoltaic applications due to their nontoxicity, high absorption coefficient, favorable bandgap for capturing solar photons, and global elemental availability.^12,13^ However, similar to the case of PbS QDs, ABS QD-derived thin films suffer from sluggish charge migration due to the presence of long-chain organic ligands, such as oleic acid (OA), on their surfaces, as well as the small exciton Bohr radius (∼4.6 nm).^14,15^ To address the former issue, a solid-state ligand-exchange process has been recently implemented during film formation using a layer-by-layer approach.^16−18^ In addition to the replacement of insulating organic ligands, this process can be used to favorably tune the band energies for improved alignment with charge selective contact layers, thus promoting photocarrier separation and extraction. For instance, Lee et al. generated iodide- and thiolate-terminated ABS QDs using solutions of tetramethylammonium iodide (TMAI) in methanol and 2-mercaptoethanol (2-ME) in acetonitrile to tune ABS Fermi energy levels. The resulting film, containing ABS QDs capped by organic/inorganic (2-ME/TMAI) ligands, achieved a PCE of 7.1% due to improved hole transport resulting from energy-level alignment between ABS and the hole transporting layer (HTL).^19^ In addition, Choi and co-workers modified the energy levels of ABS QDs via the ligand exchange method and put forward a hard–soft acid–base theory to explain the binding preferences between metal cations and functional groups.^18^ Using this model, they rationalized the *p*-type nature of ABS following treatment by 3-mercaptopropionic acid (MPA). However, a systematic methodology for regulating the electronic properties of ABS QDs and their surfaces, which is crucial for designing efficient junctions, remains in its infancy. Thus, while ABS-based photovoltaic devices continue to suffer considerable losses associated with poor carrier transport, there remain considerable opportunities for performance improvement by electronic structure and energy level engineering.

In this work, we demonstrate a strategy to systematically tailor band edge and Fermi level positions via a solvent-induced ligand exchange (SILE) approach. To eliminate the OA ligand on as-synthesized QDs, pristine ABS films (denoted as ABS-OA) were treated with various MPA-methanol (MPA-MeOH) solutions. The concentration of MPA in the solution was varied to tune the quantity of ligand molecules bound to the surface. The as-prepared ABS films exhibit *n*-type character for MPA concentrations exceeding 5 vol % (denoted as ABS-MPA), which is attributed to the abundance of electron-donating thiol groups of MPA bound to the NC surfaces. However, ABS films exhibit *p*-type character when the concentration of MPA in MeOH is reduced to zero (denoted as ABS-MeOH). Based on photoemission spectroscopy, we attribute this *p*-type conductivity to interactions of ligand-free QDs with atmospheric oxygen, which serves as an electron acceptor. By systematically comparing the impacts of a broad range of different solvents and ligand-solvent solutions, we discovered that only polar solvents induce *p*-type doping and that this behavior depends on the ligand content within those polar solvents. Furthermore, we show that this behavior is reversible by application of sequential ligand-solvent treatments, is compatible with both organic and inorganic ligands, and can be utilized with both electron donating and electron withdrawing molecular groups. Based on these findings, we used the SILE approach to design and fabricate ultrathin ABS solar cells featuring *p*–*n* heterojunction architectures with optimized band alignment, yielding a notable PCE of 7.43%. This work thus provides valuable insights for regulating electronic properties of advanced semiconductor films for photovoltaic applications.

## Results and Discussion

**Figure 1:**
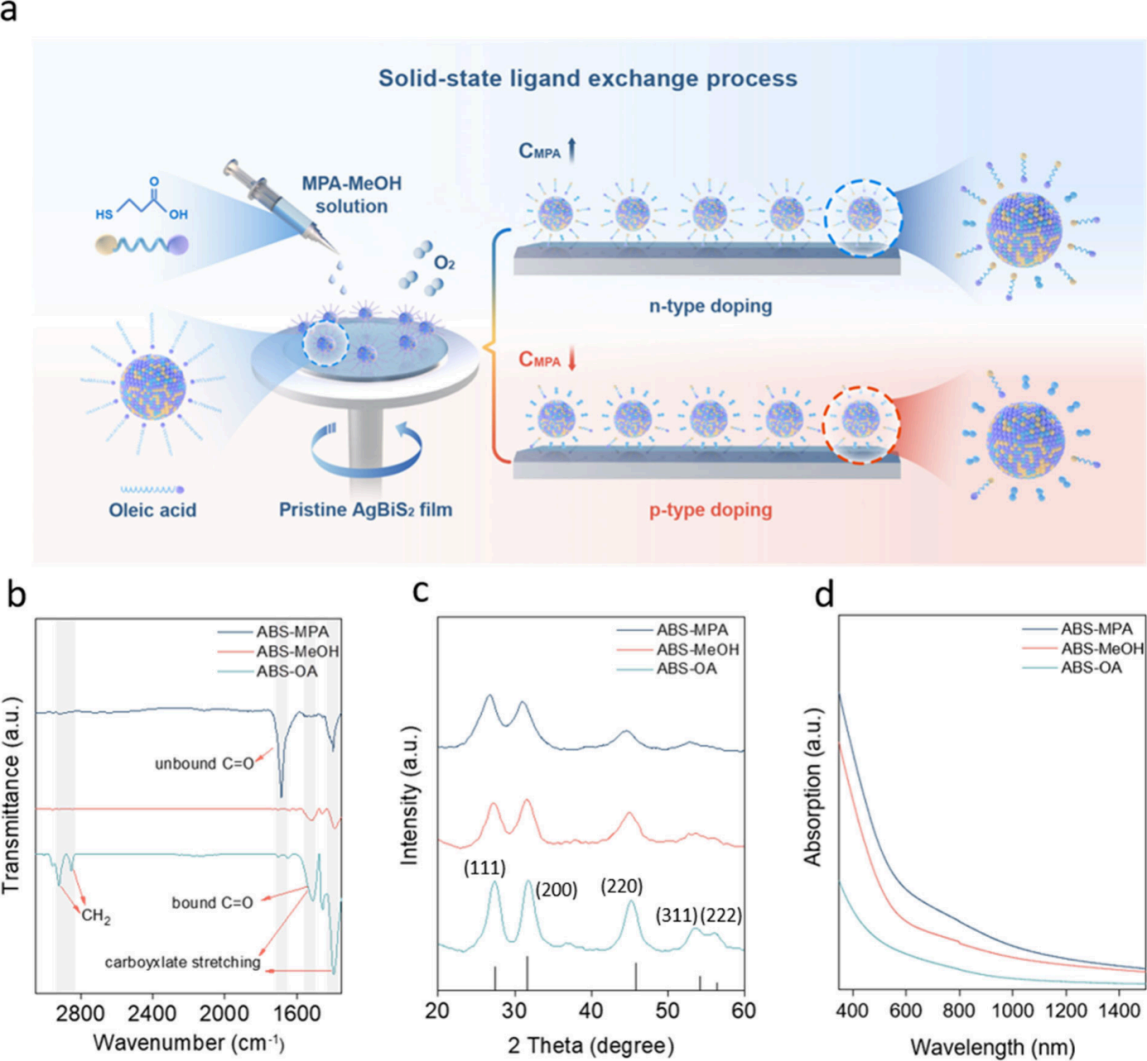
Engineering ABS band alignment. (a) Schematic of the SILE process and controllable doping mechanism. (b) FTIR spectra of ABS-OA, ABS-MeOH, and ABS-MPA. (c) XRD patterns of ABS-OA, ABS-MeOH, and ABS-MPA. The corresponding reference is shown as black bars (PDF #21-1178). (d) UV–vis absorption spectra of ABS-OA, ABS-MeOH, and ABS-MPA.

Dispersions of pristine ABS QDs capped by OA ligands were synthesized using the hot-injection method, as previously reported by Bernechea et al.,^12^ after which thin films were deposited onto planar substrates via the layer-by-layer approach.^13^ Using these approximately 30 nm thick films, we applied a solid-state ligand exchange approach to investigate the roles of various solvent and ligand-solvent solution treatments on the electronic properties of ABS, demonstrating that the majority carrier type can be controllably modulated between *n*- and *p*-type. Overall, this SILE approach represents a powerful, yet straightforward, method for tuning the electronic properties and energetic levels of ABS QDs. Figure 1a shows a schematic depiction of the SILE method and the hypothesized doping mechanism, which is described in detail and experimentally verified below. In brief, the initially OA-capped ABS QDs were treated with MeOH solutions containing different concentrations of MPA. During this treatment, OA ligands are first eliminated by the polar solvent MeOH, after which the electron-donating thiol group and electron-withdrawing carboxyl group from the MPA ligand bind to Ag and Bi unpassivated sites, respectively.^18^ However, as the MPA concentration is decreased, the ligand coverage also decreases and unpassivated surface sites are generated. Similar to previous observations of halide perovskites,^20−22^ exposure to air results in adsorption of molecular oxygen at unpassivated surface sites, with the electron withdrawing nature of the bonded oxygen inducing *p*-type doping of the QDs. In this way, the SILE approach provides a straightforward means of tuning the electronic properties of ABS nanocrystals, while also reducing interparticle transport resistances associated with long organic ligands.

Electronic transport measurements of ABS films deposited on insulating silica substrates confirm that, compared with as-deposited OA-capped layers, the resistivities decrease by up to 2 orders of magnitude after both MeOH and MPA-MeOH treatments, as expected for the removal of long insulating OA ligands (Table S1).^3^ In addition, the carrier mobilities increase from 11.5 cm^2^/V·s to 14.3 and 15.5 cm^2^/V·s, respectively, after these surface treatments. To directly verify that the OA ligands were removed by the SILE approach, Fourier transform infrared spectroscopy (FTIR) was performed (Figure 1b). Following MeOH treatment, the distinctive vibrational modes of the −CH_2_– group at 2924 and 2852 cm^–1^ vanished. This change was accompanied by a substantial decrease in the intensity of the −COOH modes at 1420 and 1550 cm^–1^, thus confirming significant elimination of OA ligands.^14,23,24^ For ABS-MPA, the presence of faint signals attributed to −CH_2_– groups and peaks corresponding to −COOH indicate the occupation of unpassivated sites on the ABS surface by MPA molecules. Furthermore, the distinct signal assigned to unbound C=O stretching vibrations at 1700 cm^–1^, the weak signals of bound C=O vibrations at 1550 cm^–1^, and the near complete absence of S–H stretching modes at 2550 cm^–1^ confirm that MPA primarily binds to the unpassivated sites via the thiol group, thereby explaining the tendency of MPA to induce *n*-type doping.^14^

To investigate the impact of terminal surface groups on ABS film morphology, scanning electron microscopy (SEM) at various magnifications was performed before and after each treatment (Figure S1). After methanol exposure, ABS QDs displayed a tendency to aggregate, attributed to the removal of oleic acid (OA) ligands and the presence of nonpassivated sites, leading to the formation of large ABS particles (Figure S1d–f). However, as the concentration of MPA increased, this aggregation was progressively reduced (Figure S1g–i). At 1 vol % MPA, only minor clustering was observed, highlighted by the red circle in Figure S1h,i. At 5 vol % MPA, the ABS films exhibited a much more uniform morphology, indicating improved QD dispersion, superior film quality, and effective passivation (Figure S1j–l). Additionally, transmission electron microscopy (TEM) was used to observe the changes in ABS QD dispersion before and after methanol treatment, as shown in Figure S2. Consistent with the SEM findings, the TEM images confirmed that ABS QDs tend to aggregate due to the presence of nonpassivated sites following methanol treatment, indicating the important role of surface ligands on film morphology. X-ray diffraction (XRD) analysis confirmed that the particles maintain their expected cubic rock salt structure; however, a reduction in crystallinity was observed, as evidenced by the increased full width at half-maximum (fwhm) of all XRD peaks following solid-state ligand exchange (Figure 1c). Additionally, atomic force microscopy (AFM) images (Figure S3) revealed an increase in film roughness after methanol treatment, which can be attributed to the aggregation of QDs. UV–vis absorption measurements reveal the expected broad spectral absorption of ABS, which extends across the visible range and into the near-infrared region (Figure 1d), with Tauc analysis indicating a direct bandgap (*E*_g_) of ∼1.25 and ∼1.18 eV for the MeOH and MPA-MeOH treated films (Figure S4), respectively. Importantly, comparison of absorption spectra obtained immediately after MeOH treatment and after 10 days of exposure to ambient air reveals minimal changes of the optical properties of ABS films (Figure S5), suggesting their high stability and potential suitability for use in robust devices, despite the absence of long-chain organic capping ligands.

**Figure 2:**
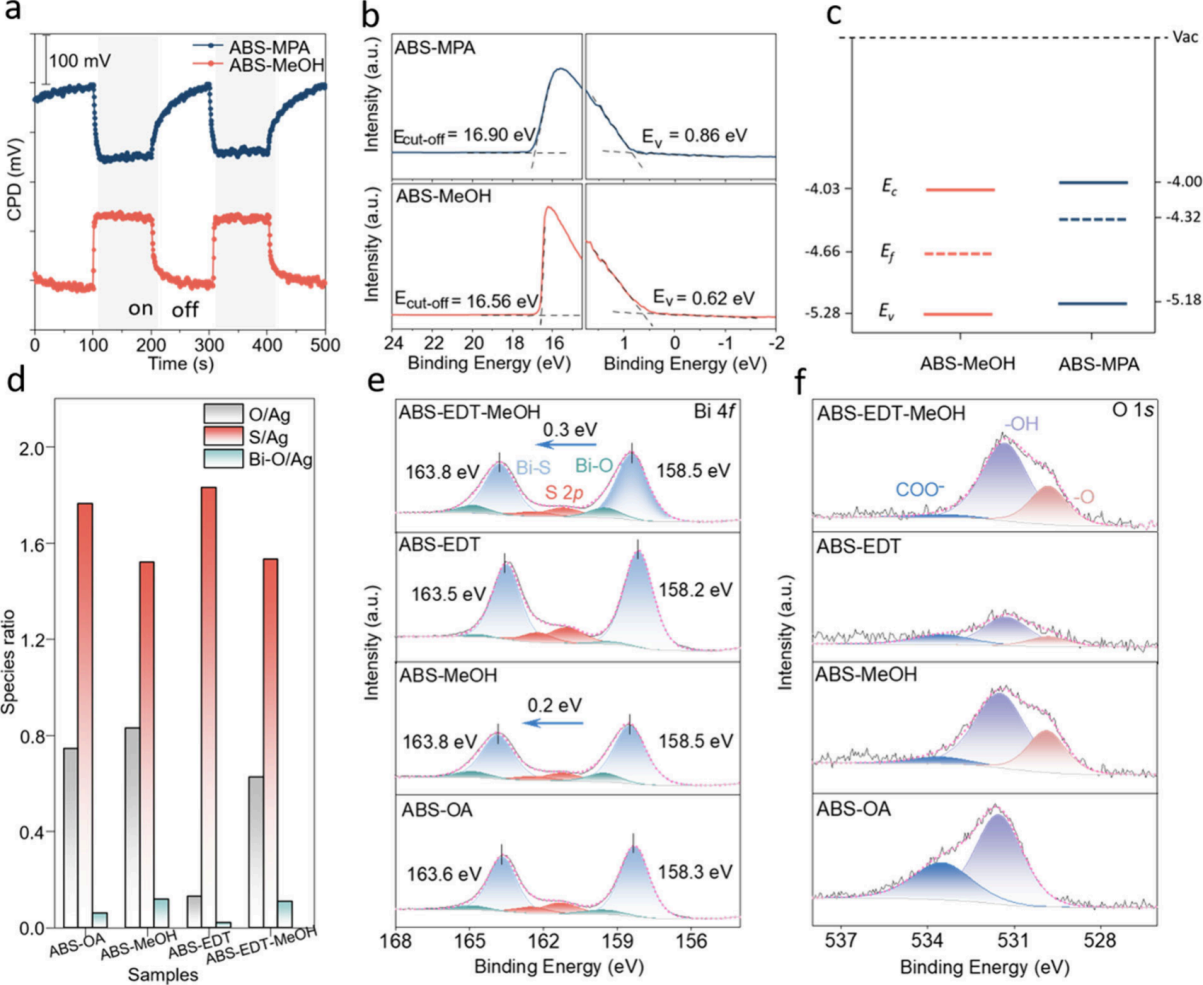
Changes of ABS surface chemistry after ligand exchange. (a) Change of CPD as a function of time in the dark and under 455 nm LED illumination for ABS-MPA and ABS-MeOH, with the gray areas indicating the times during illumination. (b) UPS results for ABS-MPA and ABS-MeOH. (c) Energy levels with respect to vacuum for ABS-MeOH and ABS-MPA. (d) Atomic ratios of O/Ag (gray) and S/Ag (red), as well as the component of Bi–O/Ag (green), calculated from XPS analysis of the O 1*s*, S 2*p*, Bi 4*f* Bi–O component, and Ag 3*d* core level spectra for ABS, ABS-MeOH, ABS-EDT, and ABS-EDT-MeOH. (e) Corresponding XPS Bi 4*f* and S 2*p* core level spectra, including the fitted components. (f) Corresponding XPS core level spectrum O 1*s*, including fitted components.

To assess the impact of the SILE process on the electronic properties of ABS films, we performed surface photovoltage (SPV) measurements using the contact potential difference (CPD) method with periodic 455 nm LED illumination under ambient conditions. In brief, CPD method for detecting the SPV of a given semiconductor involves measuring the differences in work functions between a metallic reference probe and the semiconductor surface in darkness compared to during illumination. This CPD itself is given by *V*_CPD_ = (φ_ref_ – φ_sc_)/*e*, where φ_ref_ and φ_sc_ represent the work function of the reference probe (here Au) and semiconductor, respectively.^25^ The SPV can be calculated according to the formula: SPV = CPD_light_ – CPD_dark_.^26^ When illuminated, the quasi-Fermi level splitting in a *p*-type material typically leads to an increase of φ_sc_ and thus a positive SPV. Conversely, for an *n-*type semiconductor, the effect is opposite. Therefore, the SPV can serve as an indicator for the majority carrier type. As shown in Figure 2a, a positive SPV was observed following treatment in pure MeOH, suggesting *p*-type doping of the film. Conversely, treatment of the ABS film in an MPA-MeOH solution resulted in a negative SPV, which is suggestive of *n*-type doping. Consistent with these SPV results, complementary Hall effect measurements (Table S1) confirm the *p*- and *n*-type character of MeOH and MPA-MeOH treated films, respectively. Here, we note that SPV quantifies the magnitude and direction of the surface band bending rather than the carrier type directly. However, upward (downward) band bending is typical for *n*-type (*p*-type) semiconductors. Here, the correspondence between SPV and Hall results confirms that this measurement provides an effective, noninvasive proxy for determining the carrier type in ABS thin films.

Ultraviolet photoelectron spectroscopy (UPS) measurements provide important insights into the impact of ligand exchange on the band edge and surface Fermi level energetics. As shown in Figure 2b, the MeOH treated film displayed a larger work function of 4.66 eV compared to the value of 4.32 eV observed for the MPA-MeOH treated ABS. Likewise, the valence band positions (*E*_v_) for MeOH and MPA-MeOH treated ABS films were determined to be −0.62 and −0.86 eV relative to the Fermi level, respectively. Considering the bandgaps of the ABS films discussed above, the conduction band positions (*E*_*c*_) can also be established, as shown in Figure 2c. While pure MeOH treatment results in a slight downward shift of the band positions relative to the MPA-MeOH sample, the impact on the Fermi level (*E*_*f*_) is much more pronounced. In particular, the ∼320 meV downward shift of *E*_*f*_ is consistent with its change from *n*-type (MPA-MeOH) to weakly *p*-type (MeOH) character. As discussed above, we hypothesize that the higher concentration of adsorbed oxygen at unpassivated surface sites of MeOH-treated ABS is responsible for its *p*-type nature. In this regard, it is important to note that the adsorbed surface oxygen coverage during ultrahigh vacuum (UHV) UPS measurements is likely lower than during SPV measurements in ambient air. Thus, the UPS-determined change of the Fermi level position may underestimate the magnitude of its downward shift.

X-ray photoelectron spectroscopy (XPS) was next performed to probe changes in the composition and binding environment following SILE treatments. To enable detailed analysis of the role of oxygen on the observed behavior, we first performed a solid-state ligand exchange using EDT, which is an oxygen-free molecule, unlike OA and MPA. Thus, by comparing OA- and EDT-capped ABS before (ABS-OA and ABS-EDT, respectively) and after MeOH treatment (ABS-MeOH and ABS-EDT-MeOH, respectively), it is possible to better understand the role of adsorbed oxygen on the surface chemistry and semiconductor properties of ABS. Figure 2d summarizes the XPS-derived peak intensity ratios of O(1*s*)/Ag(3*d*), Bi(4*f*)-O/Ag(3*d*), and S(2*p*)/Ag(3*d*) from each of the samples before and after MeOH treatment, where the Bi(4*f*)-O intensity is given by the deconvoluted spectral contribution associated with Bi–O binding (Figure 2e). As expected, replacement of OA with EDT leads to a significant decrease of the O content, as well as an increase of S due to adsorption of thiol groups of EDT ligands onto ABS (ABS-EDT). While MeOH treatment leads to an increase of oxygen for both ligands, the effect is significantly more pronounced for the case of ABS-EDT-MeOH due to the absence of ligand oxygen in the starting ABS-EDT sample. For both ABS-MeOH and ABS-EDT-MeOH, the increased oxygen content is consistent with oxygen adsorption at unpassivated surface sites. This conclusion is supported by closer inspection of the Bi 4*f* and O 1*s* core level region in Figure 2e,f, which reveals an increase of the metal–O bonding component at the surfaces of the ABS films following MeOH treatment. According to high-resolution Bi 4f spectra in Figure 2e, we observe a blue shift of 0.2 and 0.3 eV after MeOH treatment for Bi–S peaks in ABS-MeOH and ABS-EDT-MeOH, respectively. This shift is also evident in the high-resolution Ag 3d spectra shown in Figure S6, indicating a decrease in electron density with adsorbed oxygen content, corresponding to the *p-*type doping state.^12^

In order to further probe the influence of adsorbed oxygen on the electronic properties of ABS films, we compared CPD measurements for the case of MeOH treatments performed in an air-free glovebox to those conducted in ambient air. As presented in Figure S7, the magnitude of the SPV increased from 60 mV (air-free glovebox) to 140 mV (ambient air), suggesting enhanced *p*-type character for ABS with increased oxygen exposure. Moreover, we also performed CPD measurements in an N_2_ environment following MeOH treatments in an air-free glovebox to minimize contact with oxygen. As presented in Figure S8, a negative SPV that is consistent with *n-*type character was observed, indicating that absorbed oxygen plays an important role on the electronic properties of ABS. We note that CPD measurements of ABS treated with water (Figure S9) strongly suggest that water in air is not responsible for the *p*-type doping of ABS.

**Figure 3:**
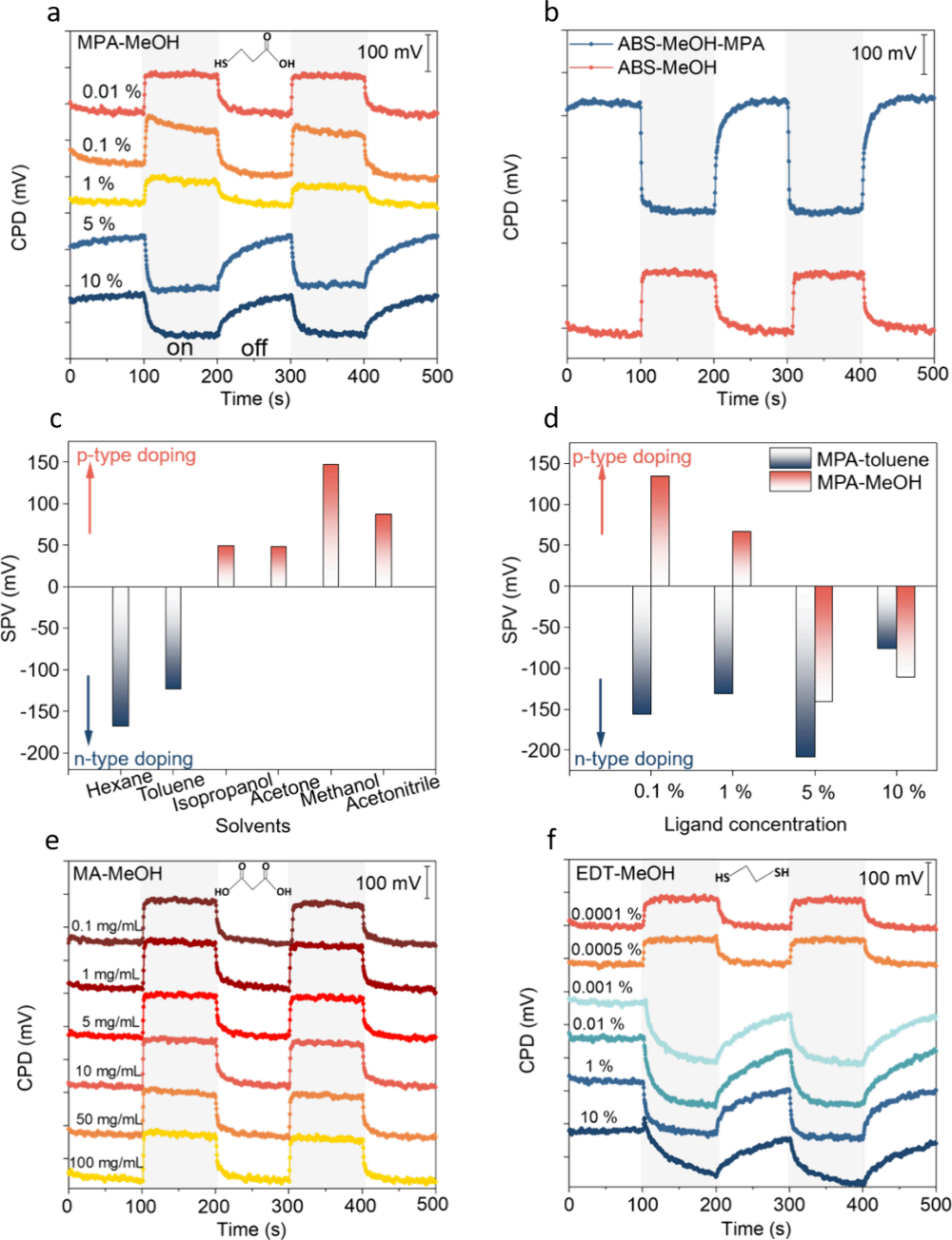
Effects of solvents and ligands on CPD changes of ABS. (a) Change of CPD as a function of time for ABS treated by MPA-MeOH solution with varied concentration in the dark and under illumination. The inset shows the structure of MPA. (b) Change of CPD as a function of time for ABS-MeOH and ABS-MeOH-MPA in the dark and under illumination. (c) CPD changes for ABS treated by different solvents with varied polarity. (d) CPD changes for ABS treated by MPA ligand dissolved in MeOH and toluene with varied concentration. (e) Change of CPD as a function of time for ABS treated by MA-MeOH solution with varied concentration. The inset shows the structure of MA. (f) Change of CPD as a function of time for ABS treated by EDT-MeOH solution with varied concentration in the dark and under illumination. The inset shows the structure of EDT. The gray areas in (a) and (b) and (e) and (f) indicate the times at which the samples were illuminated by the 455 nm LED.

Having established that the SILE approach allows transitions from *n*- to *p*-type doping of ABS, we next sought to understand how the ligand concentration and solvent polarity can be used to precisely control the electronic properties of the semiconductor. As a starting point, the SILE approach was applied to ABS films with varied concentrations of MPA in MeOH, ranging from 0.01 to 10 vol %. As shown by the SPV results in Figure 3a, ABS shows *p*-type character when the MPA concentration is less than 1 vol %, while *n*-type character is observed when the MPA concentration exceeds 5 vol %. This behavior is consistent with a concentration-dependent surface coverage of ligand, which results in competing contributions from electron-withdrawing oxygen adsorbates at unpassivated surface sites and electron-donating thiol groups from MPA bound to ABS. With increasing MPA concentration, increasingly compact ligand shells lead to increased donation of electrons from thiol groups and reduced concentrations of unpassivated sites for oxygen to adsorb, resulting in *n*-type character. This interpretation is supported by the results displayed in Figure 3b, which shows a MeOH-treated sample initially possessing the expected *p*-type character that was subsequently treated with a high concentration (5 vol %) MPA solution, resulting in the recovery of *n*-type behavior. Importantly, this finding indicates that the SILE strategy can be used to reversibly tune the doping type in ABS films, from *n*-type to *p*-type, and back again, as discussed in greater detail below.

In addition to tuning electronic properties of ABS via the ligand concentration, we also explored the role of the solvent itself. To this end, we treated ABS films using several different solvents with varying polarity, ranging from acetonitrile with a dielectric constant ε = 37.5 to hexane with ε = 2.02 (Table S2). Figure 3c illustrates that only polar solvents are capable of inducing *p*-type doping, as indicated by the positive SPV, since their large dielectric constants can effectively screen attractive interactions between ABS and OA molecules, leading to detachment and dissolution of the organic ligands.^27^ For this reason, regardless of the ligand concentration, ABS treated by MPA dissolved in nonpolar solvents, such as toluene or hexane, leads to preservation of the *n*-type doping state (Figure 3d). Based on these collective observations, we conclude that the SILE strategy can be used to broadly tune doping of ABS, and thus its Fermi energy, by controlling the concentration and type of surface adsorbates with rationally selected ligand-solvent solutions with specified dielectric constants. Overall, choosing polar solvents is crucial for achieving *p*-type conductivity, with more pronounced effects for decreasing concentrations of electron donating ligands in solution and with increasing solvent dielectric constant.

To further verify the versatility of the SILE strategy for achieving *p*- or *n*-type doping, additional measurements were conducted for ABS decorated by three other types of ligands–both organic and inorganic–featuring different terminal groups. First, we utilized malonic acid (MA), which contains two carboxyl end-groups and has a strong ability to withdraw electrons, thus inducing a *p*-type behavior in ABS. Consistent with expectations, we find that solid-state ligand exchange using MA in MeOH always results in a positive SPV, indicative of *p*-type character, regardless of the MA concentration (Figure 3e). Second, we also explored the concentration-dependent behavior of EDT ligands, which possess two electron donating thiol groups. In this case, we observe a negative SPV, indicative of *n*-type character, for all but the lowest concentrations of EDT in MeOH (Figure 3f). In particular, a transition to *p*-type behavior is only observed when the EDT concentration is <0.0005 vol %, which is substantially lower than for the case of MPA. While both MPA and EDT can bind to ABS via electron-donating thiol groups, a fraction of MPA can also bind via the electron withdrawing carboxyl end-group, as schematically represented in Figure 1a. Thus, EDT provides a greater overall electron donating capability for a given ligand coverage, which can contribute to the observed difference in critical concentration below which *p*-type behavior is observed. Finally, beyond organic ligands, we also explored the compatibility of this method with inorganic ligands, such as I^–^. As shown in Figure S10, ABS treated with TMAI-MeOH solution exhibits a concentration-dependence that is similar to that of EDT due to the electron-donating character of I^–^, indicating that the SILE strategy applies to organic and inorganic ligands alike.

As mentioned above, sequential application of the SILE approach enables reversible changes of the doping type. To further illustrate this point using a diversity of different ligand types, we performed a series of successive SILE treatments and probed the changes of doping type via SPV. As shown in Figure S11, ABS pretreated with 0.01 vol % EDT solution with *n*-type character can be switched to *p*-type behavior following treatment with a MeOH solution containing 10 mg/mL MA. Likewise, ABS pretreated with 10 mg/mL of MA in MeOH is *p*-type and can be converted to *n*-type character following treatment with a solution containing 0.01 vol % EDT. Thus, we conclude that the SILE approach provides a straightforward and versatile means of tuning the electronic properties of ABS, offering compatibility with both organic and inorganic ligands featuring either electron-withdrawing or electron-donating characteristics.

**Figure 4:**
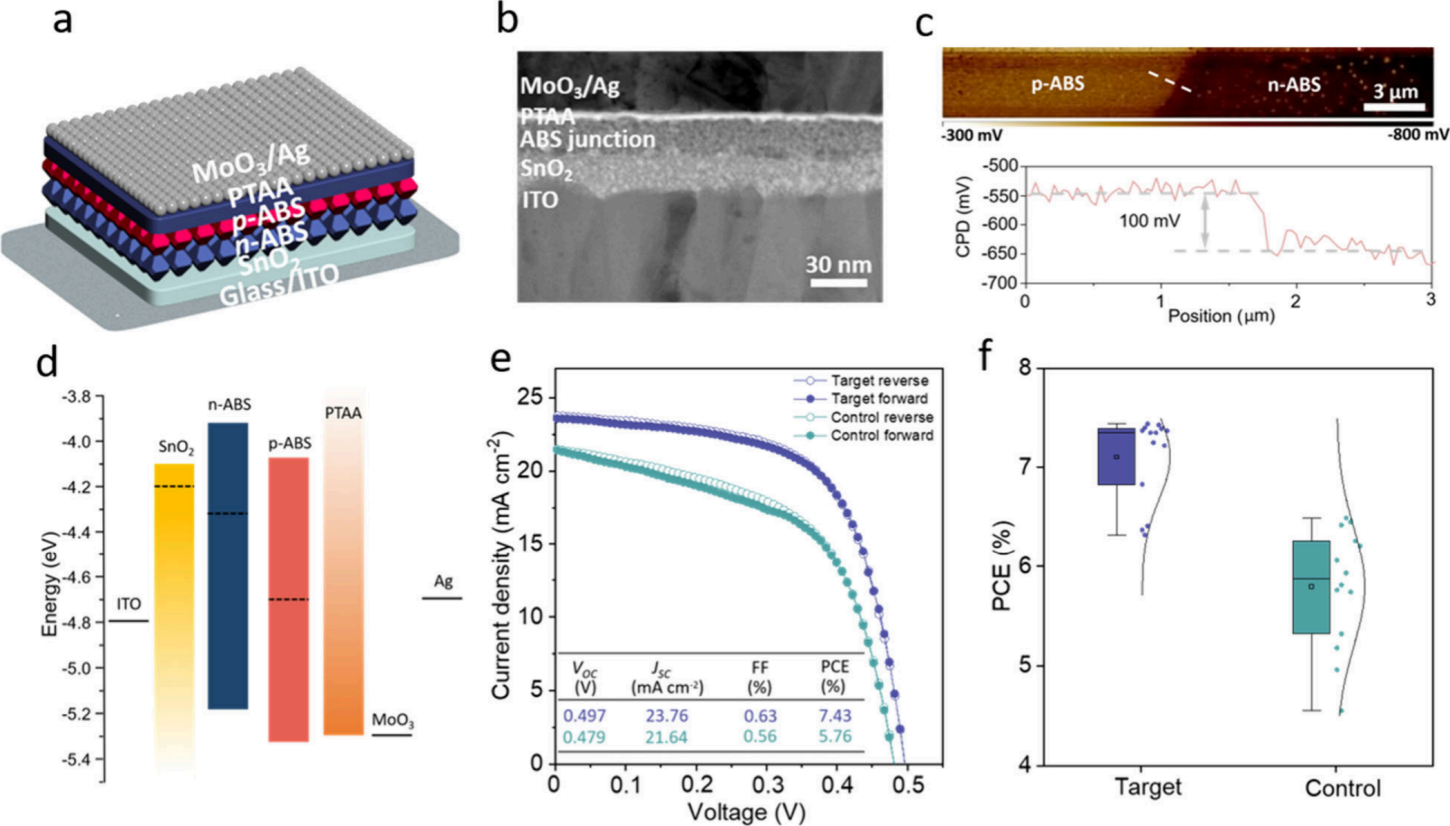
Performance of photovoltaic cells comprising ultrathin ABS films. (a) Schematic of the target device structure composed of a first layer of ABS-5 vol % MPA and second layer of ABS-1 vol % MPA. (b) Cross-sectional HAADF-STEM image of the target device. (c) KPFM images of a reference lateral *p*–*n* heterojunction and the corresponding line profile of the potential across the heterojunction edge under illumination. (d) Comparison of band edge and Fermi level position of each of the layers comprising the device with optimized performance. (e) *J*–*V* curves of the champion device of target sample (ABS *p*–*n* heterojunction) and control sample (ABS-1 vol % MPA). (f) PCE statistics of target and control devices represented in box-and-whisker plots.

Based on the findings described above, we next sought to exploit the SILE method to create photovoltaic devices featuring internal *p-n* heterojunctions. Here, we note that although both sides of the junction comprise ABS, they should be considered heterojunctions due to the changing band edge positions and differing ligand compositions in the *p*- and *n*-type regions. As described in the Experimental Section and illustrated in Figure 4a, we therefore fabricated structures comprising glass/indium tin oxide (ITO)/SnO_2_/*n*-ABS/*p*-ABS/PTAA/MoO_3_/Ag layer stacks (denoted as the target device), with PTAA as hole transport layer (HTL). To verify that the underlying *n*-ABS layer retains its *n*-type character after application of the SILE process that is used to generate *p*-ABS overlayer, reference measurements were performed on *n*-ABS films treated with pure MeOH treatment to simulate an extreme example of the SILE condition. Importantly, no significant change in SPV was observed (Figure S12), indicating that the second SILE process used to form the upper layer has negligible impact on the underlying *n*-ABS layer. We note that each layer was subjected to mild annealing following its formation, which stabilized is against additional solvent modification.

The cross-sectional high-angle annular dark-field scanning transmission electron microscopy (HAADF-STEM) image in Figure 4b demonstrates the closed and ultrathin nature of the layers, including an approximately 30 nm thick ABS absorber film. To estimate the energy alignment between *n*- and *p*-type ABS, a reference Kelvin probe force microscopy (KPFM) measurement of the change of surface potential across a reference planar lateral ABS *p*-*n* heterojunction was performed. As depicted in Figure 4c, a surface potential difference of ∼100 mV between *p*-ABS (produced from 1 vol % MPA in MeOH) and *n*-ABS (produced from 5 vol % MPA in MeOH) was observed, indicating the formation of a built-in electric field oriented from *n*-ABS to *p*-ABS that can promote charge carrier separation to the respective selective contact layers within solar cell structures. The band positions of each of the layers are shown in Figure 4d, with energy levels for the *n*-ABS and *p*-ABS determined by the UPS measurements (Figures 2b and S13) and those of the charge-extracting layers and electrodes given by previous reports.^12,14^ Here, we note that measurements of absolute band positions can be subject to uncertainty due, for example, to the presence of surface adsorbates that are not present at the buried solid/solid interface. While such measurements should therefore be interpreted with caution, the band positions of the *p*-ABS and *n*-ABS layers suggest favorable alignment with the charge selective SnO_2_ and PTAA layers, respectively, as well as the formation of an internal electric field that promotes directional charge separation. While our UPS measurements imply band offsets that would lead to energetic barriers at the ABS *p*–*n* junction, we expect that improved interface energetics could be realized through tuning of ligand dipoles and chemical interactions with the surfaces of ABS particles.

As a control of the *n*-ABS/*p*-ABS junction in our device, an ITO/SnO_2_/*p*-ABS/PTAA/MoO_3_/Ag structure, with an identical total ABS thickness of 30 nm, was also fabricated. The *J*–*V* characteristics of all devices were evaluated under AM1.5G illumination. As illustrated in Figures 4e,f and S14, the target *p*–*n* heterojunction device demonstrated superior performance, with an average PCE of 7.43%, open-circuit voltage (*V*_OC_) of 0.497 V, fill factor (*FF*) of 0.63, and short-circuit current density (*J*_SC_) of 23.76 mA cm^–2^ compared to the control device, which exhibited an average PCE of 5.76%, *V*_OC_ of 0.479 V, *FF* of 0.56, and *J*_SC_ of 21.64 mA cm^–2^. The enhanced performance characteristics of the *p*–*n* heterojunction device are attributed to improved carrier separation from the built-in electric field. In addition, the target device exhibited less hysteresis than the control when the voltage was scanned in both forward and reverse modes, suggesting reduced charge trapping and a better reliability in the reported efficiency. External quantum efficiency (EQE) spectra in Figure S15 show that the target device exhibits higher *J*_SC_ compared to the control device. As additional control devices, structures composed of ITO/SnO_2_/*n*-ABS/PTAA/MoO_3_/Ag and an inverted *p*–*n* ABS stack of ITO/SnO_2_/*p*-ABS/*n*-ABS/PTAA/MoO_3_/Ag were also prepared and evaluated. As depicted in Figure S16, the former exhibited a PCE of 5.65%, *V*_OC_ of 0.469 V, *FF* of 0.58, and *J*_SC_ of 20.77 mA cm^–2^, while the latter suffered from even poorer performance, with a PCE of 4.75%, *V*_OC_ of 0.455 V, *FF* of 0.48, and *J*_SC_ of 21.58 mA cm^–2^ due to the energy level mismatch not only between SnO_2_ and *p*-ABS but also between PTAA and *n*-ABS. To further prove that a junction is formed between the two ABS layers and to eliminate the impacts of HTL and ETL, we fabricated structures comprising ITO/*n*-ABS/*p*-ABS/MoO_3_/Ag layer stacks (5 vol %+1 vol %) and ITO/*p*-ABS/MoO_3_/Ag (1 vol % + 1 vol %). Compared to the target device, the *p*–*n* stack without SnO_2_ or PTAA layers showed inferior performance, with an average PCE of 2.29%, *V*_OC_ of 0.443 V, *FF* of 0.36%, and *J*_SC_ of 14.45 mA cm^–2^. This reduced performance is attributed to poor carrier collection, highlighting the crucial role of ETL and HTL layers (Figure S17a). Moreover, in contrast to the *p*–*n* junction device, the stack composed solely of *p*-ABS exhibited an undetectable photovoltaic response (Figure S17b), which can be attributed the lack of a built-in electric field from the *p*–*n* interface,^28^ underscoring the importance of assembling the *p*–*n* heterojunction. As discussed above, UPS measurements indicate the possible presence of an in internal energetic barrier due to band offsets at the *p*–*n* junction interface. Nevertheless, the device performance characteristics reported above indicate that the net result of junction formation is to increase the PCE due to enhanced charge separation and extraction.

**Figure 5:**
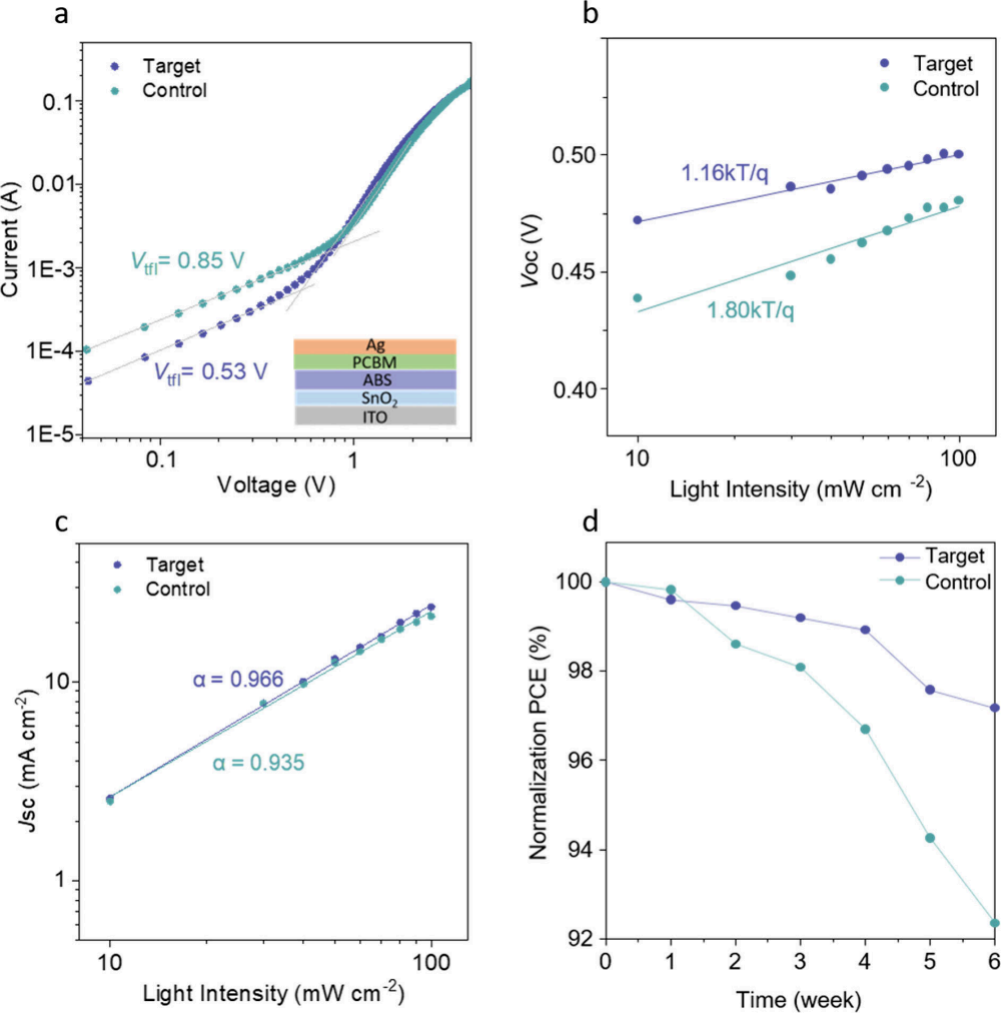
Transport, recombination, and stability of ABS-based cells. (a) SCLC curve of target and control electron-only samples. Inset shows the schematic structure of electron-only device. (b) Light-intensity-dependence of *V*_OC_. (c) Light-intensity-dependence of *J*_SC_. (d) Long-term stability of ABS-based solar cells.

To better understand the origin of the improved performance of the *p*–*n* heterojunction system, we performed additional measurements of its electronic characteristics and compared those to the *p*-type control. To this end, the space-charged-limited current (SCLC) method was employed to quantify the defect density of analogous electron-only devices, as shown in Figure 5a. From these data, the trap densities were determined according to the equation: *N*_trap_ = 2εε_0_*V*_tfl_/*eL*,^2^ where *V*_tfl_ is the turning-point voltage of the trap-filled limit region (0.53 V for the target device and 0.85 V for the control device), ε_0_ is the vacuum permittivity, ε is the relative dielectric constant of ABS, *e* is the elementary charge, and *L* is the thickness of the ABS layer in the devices.^29^ This analysis shows that the trap density decreased from 1.12 × 10^18^ cm^–3^ (control device) to 7.17 × 10^17^ cm^–3^ (target device), indicating improved optoelectronic quality of the target device. To explore the charge collection efficiency, the *V*_OC_ was measured as a function of the light intensity, as shown by the plot in Figure 5b. The decreased slope of 1.16 *kT*/*q* for the target device compared to 1.80 *kT*/*q* in the control device, where *T*, *k*, and *q* represent the absolute temperature, the Boltzmann constant, and the elementary charge, respectively, suggests suppression of trap-assisted Shockley-Read-Hall recombination in the target device.^30,31^ Furthermore, as shown in Figure 5c, the dependence of *J*_*SC*_ on light intensity was measured and fitted with a power-law expression (*J*_SC_ ∝ *I*^α^), where *I* represents the light intensity and α is the power factor.^32^ Consistent with the results presented in Figure 5b, the target device exhibits an increased power factor (α = 0.966) compared to the control device (α = 0.935), indicates superior charge extraction.^33^ Finally, we assessed the long-term stability of the devices. As presented in Figure 5d, the unencapsulated target device maintained 97.3% of its initial PCE after being stored in ambient conditions (25 °C and ∼50% RH) for 6 weeks. In comparison, the control device exhibited a more pronounced performance decline, though it retained greater than 92% of its original PCE. Taken together, these results indicate the target *p*–*n* heterojunction device exhibited superior performance due to improved carrier diffusion, separation, and extraction, which was facilitated by enhanced built-in electric fields, lower defect densities, and improved energetic alignment.

## Conclusion

In summary, we demonstrated that solvent polarity and ligand concentration during solid state ligand exchange processes can be used to manipulate the band edge and Fermi level positions in thin films derived from ABS QDs. Our results indicate that this SILE method enables controllable and reversible transitions from *n*-type to *p*-type majority carrier doping, while also eliminating long-chain insulating ligands, resulting in enhanced interparticle charge transport. The approach is compatible with both organic and inorganic ligands, as well as those offering electron donating or electron accepting groups. Importantly, oxygen adsorption at unpassivated surface sites generated after exposure to polar solvents leads to *p*-type character, even in the absence of intentionally incorporated ligands. Taking advantage of the SILE method as a straightforward approach to tune film energetics, we integrated ABS *p*–*n* heterojunctions into solar cell structures in which improved band alignment, increased charge separation, and reduced carrier trapping resulted in a photovoltaic PCE of 7.43% under 1 Sun AM1.5 illumination. Overall, this study introduces a systematic approach to manipulate the semiconductor characteristics of ABS QD-derived thin films and engineer their junctions, thereby offering a versatile route to tailor advanced semiconductor films for photovoltaic applications.

## Experimental Section

### Materials

Bismuth(III) acetate (99.999%) and silver acetate (99.99%) were purchased from Alfa Aesar. Hexamethyldisilathiane (HMS), oleic acid (OA), octadecene (ODE), tetramethylammonium iodide (TMAI, 99%), 1,2-ethanedithiol (EDT, 98%), malonic acid (MA), 3-mercaptopropionic acid (MPA), MeOH (99.9%), and toluene (anhydrous, 99.9%) were purchased from Sigma-Aldrich. Poly[bis(4-phenyl) (2,4,6-trimethylphenyl) amine] (PTAA, average *M*_n_ = 17000) was purchased from J&K. A SnO_2_ colloidal dispersion (Sn (IV) oxide, 15% in H_2_O colloidal dispersion) and [6,6]-phenyl-C61-butyric acid methyl ester (PCBM) were purchased from Nano-C Tech. All chemicals were used as-received, unless otherwise noted. For use as the substrate, indium tin oxide (ITO)-coated glass (15 Ω sq^–1^) was acquired from Advanced Technology Co., Ltd. Fluorine-doped tin oxide coated glass was acquired from Sigma-Aldrich. Insulating substrates were acquired from Epredia Netherlands B.V.

### Synthesis of ABS QDs and Formation of Thin Films

ABS QDs were synthesized via the hot injection method.^12^ In particular, 2 mmol bismuth(III) acetate and 1.6 mmol silver acetate were dissolved in a mixture of 12 mL oleic acid and 7.5 mL 1-octadecene, after which the solution was held at 100 °C for 1 h while being vacuum pumped to remove oxygen and moisture. Then, 0.5 mL of hexamethyldisilathiane dissolved in 3 mL of 1-octadecene was quickly injected into the flask under N_2_ atmosphere. After a waiting time of 5 s, the heating element was removed and the reaction vessel was rapidly cooled in an ice bath for ∼5 min. The flask was then held at room temperature and was continuously stirred for 1 h. The QDs were subsequently washed with a mixture of acetone and toluene (2,1 v/v), followed by repeated centrifugation at 4000 rpm for 5 min for a total of three times. Finally, the obtained ABS nanocrystal powder was dispersed in anhydrous toluene (50 mg/mL) and stored in darkness.

Prior to forming thin films of ABS QDs, FTO substrates were cleaned by ultrasonication in acetone, isopropanol, and deionized water for 10 min each, after which they were dried with flowing N_2_. Thereafter, ABS QDs dispersed in toluene solution (25 mg/mL) were deposited onto the FTO substrate via the layer-by-layer method, with two deposition cycles used to create the final film thickness.^13^ For each layer, 50 μL of ABS solution was dropped on FTO, followed by dynamic spin-coating treatment at 2000 r.p.m for 1 min. All steps for film formation were performed in ambient air.

### Solid-State Ligand Exchange and MeOH Treatment Processes

To replace the oleate ligands of as-synthesized ABS films, the solid-state ligand exchange approach was utilized. In particular, different ligands (EDT, MA, TMAI, MPA) were first dissolved in MeOH with various concentrations, as specified in the text, and were dropped onto the ABS film and allowed to stand for 45 s, followed by cleaning with MeOH twice and with toluene once during dynamic spin-coating at 2000 r.p.m. for 2 min, after which the substrate was dried under flowing N_2_ and stored in a N_2_-filled glovebox. For the MeOH treatment, the as-deposited ABS films were directly rinsed twice with MeOH and once with toluene during dynamic spin-coating at 2000 r.p.m. for 2 min, after which the films were stored in glovebox.

### Materials Characterization

Scanning electron microscopy (SEM) was conducted on a Zeiss NVision40 FIB-SEM system to analyze changes in the morphology of ABS films before and after ligand exchange. Transmission electron microscopy (TEM) images were acquired by FEI Talos F200X instrument. To determine any associated changes of phase and crystallinity, X-ray diffraction (XRD) was performed using a desktop diffractometer (D2 PHASER, Bruker) with a Cu Kα source (λ = 1.54056 Å). We utilized the tapping mode of atomic force microscope (AFM) (Bruker MultiMode 8 System) to characterize ABS films. Ultraviolet–visible (UV–vis) absorption spectra were collected using an Agilent Technologies Cary-5000 system. Fourier transform infrared spectroscopy (FTIR) data were recorded on a VERTEX 70v spectrometer from Bruker Optics utilizing a VariGATR (Harrick) accessory. Measurements were performed in an evacuated spectrometer while the functionalized surface of the sample was pressed on the silicon ATR internal reflection element. A clean FTO/glass substrate served as reference. The reported spectra were acquired with a 4 cm^–1^ resolution and at averaged over 256 single spectra.

Contact potential difference (CPD) and surface photovoltage (SPV) measurements were performed using a KP020 K probe system from KP Technology with periodic LED illumination at a wavelength of 455 nm. To exclude the possibility that the response to illumination comes from substrate or ligand molecules, we performed reference SPV measurements on bare FTO with different ligands. As shown in Figure S18, no SPV signals are observed in the absence of ABS, confirming that the response to illumination originates from the semiconductor layer. In addition, we performed reference measurements on *n*-type TiO_2_ thin films. As shown in Figure S19, a negative SPV was observed after LED illumination, which is consistent with the expected result for an *n*-type semiconductor.

X-ray photoelectron spectroscopy (XPS) was performed on a SPECS system equipped with a monochromatized Al Kα source (*hν* = 1486.6 eV). XPS binding energies were calibrated using the C 1s core level position of 284.8 eV as a reference. Ultraviolet photoelectron spectroscopy (UPS) measurements were performed with a He–I UV-light source (21.22 eV) and a −5.0 V sample bias, which was then subtracted to determine the absolute binding energy.

For Hall effect measurements, ABS films were fabricated on insulating fused silica substrates. The Ohmic contacts (20 nm Ti as adhesive layer and 80 nm Au on top) were evaporated on top of the layers with a shadow mask with van der Pauw geometry. The samples were clamped on a sample card with prober pin and inserted into the Hall system of LakeShore Model 8404. The sample was connected to a Keithley Model 6514 system electrometer. Hall effect measurements were performed at room temperature in DC mode with an applied magnetic field of 0.9T.

Amplitude modulated Kelvin probe force microscopy (KPFM) was performed using a Bruker Dimension Icon atomic force microscope (AFM) and SCM-PIT V2 Pt–Ir-coated probes for enhanced conduction. The topography was measured at the first cantilever resonance near 65 kHz, while the CPD signal was obtained simultaneously at a second higher frequency of approximately 405 kHz. The frequency division minimizes the crosstalk between topography and CPD and thereby increases sensitivity. The electrostatic force was generated by applying an AC voltage of ∼1.5 V.

### Photovoltaic Device Fabrication

ITO-coated glass substrates underwent a cleaning process involving ultrasonication in soapy water, acetone, and isopropanol for 10 min each, followed by drying with flowing N_2_. Subsequently, the substrates were subjected to UV/ozone treatment (BZD250-S) for 30 min. The SnO_2_ electron transport layer (ETL) was then spin-cast from a diluted α-SnO_2_ colloid solution (1:2 v/v with H_2_O) at a spin speed of 2000 r.p.m., after which it was annealed at 270 °C for 30 min in air. Following formation of the ETL, two layers of ABS QDs were deposited from a 20 mg mL^–1^ toluene solution using the layer-by-layer method described above. For the first layer, 50 μL of ABS solution was spin coated on ITO/SnO_2_ at a speed of 2000 r.p.m. Then, MPA dissolved in MeOH (1:20 v/v) was dropped to the ABS film and allowed to stand for 45 s, followed by cleaning with MeOH twice and with toluene once during dynamic spin-coating at 2000 r.p.m for 2 min, after which the substrate was dried under flowing N_2_ and annealed at 115 °C for 10 min in air. The second layer of ABS was deposited using a similar procedure, but was treated with a dilute MPA MeOH solution (1:100 v/v). The resulting films were annealed at 115 °C for 10 min in air and then stored in dry air before spin-coating a PTAA solution (2 mg mL^–1^ in toluene) at 3000 r.p.m. Finally, a Kurt J. Lesker NANO 36 system was utilized to deposit a 3 nm thick MoO_3_ layer and a 120 nm thick Ag layer through a shadow mask, resulting in the fabrication of solar cells, each with a diameter of 2 mm (area: 3.14 mm^2^).

### Photovoltaic Device Characterization

The current density–voltage (*J*–*V*) curves of the ABS solar cells were measured using a 2400 Series Source Meter from Keithley Instruments. To minimize the mismatch between the simulated and actual solar spectrum to less than 2%, we employed a 150 W Class AAA solar simulator (XES-40S1, SAN-EI) as the simulated AM 1.5G standard light source for photovoltaic measurements. Prior to the test, the light intensity was calibrated using a standard monocrystalline silicon diode with a KG-5 filter. Throughout the measurement, a black-colored metal mask defined the photoactive area as 0.031 cm^2^, ensuring the accuracy of the short-circuit current density (*J*_SC_) obtained from *J*–*V* curves. The sweeping conditions comprised a reverse scan (0.55 V to −0.02 V, scan rate 10 mV s^–1^, and no delay time) and a forward scan (−0.02 to 0.55 V, scan rate 10 mV s^–1^, and no delay time). EQE spectra were measured with a QE-R3011 measurement system (Enli Technology, lnc). All measurements were conducted in ambient air at room temperature with no additional encapsulation.

## Supporting Information Available

Hall effect results table; SEM and AFM images; Tauc plots; UV–vis spectra; XPS spectra; CPD– and SPV–time traces; Solvent dielectric constants table; UPS measurements; Solar cell performance characteristics statistics; EQE spectra; Photovoltaic *J*–*V* curves (PDF)
