# Supplementary material for: Ligand-Tuned AgBiS2 Planar Heterojunctions Enable Efficient Ultrathin Solar Cells
Source: ACS Nano. 2024 Nov 27;18(49):33348–58. doi: 10.1021/acsnano.4c07621 (PMC11636261; doi:10.1021/acsnano.4c07621)
Supplement: Supplementary file 1 — nn4c07621_si_001.pdf [file nn4c07621_si_001.pdf]

Supporting Information

Ligand-tuned $AgBiS_2$ planar heterojunctions enable efficient ultrathin solar cells

Jianian Chen[1,2†], Qixuan Zhong[3†], Elise Sirotti[1,2], Guanda Zhou[1,2], Lukas Wolz[1,2], Verena Streibel[1,2], Johannes Dittloff[1,2], Johanna Eichhorn[1,2], Yongqiang Ji[3], Lichen Zhao[3], Rui Zhu[3,4,5]* and Ian D. Sharp[1,2]*

[1] *Walter Schottky Institute, Technical University of Munich, Am Coulombwall 4, 85748 Garching, Germany*

[2] *Physics Department, TUM School of Natural Sciences, Technical University of Munich, Am Coulombwall 4, 85748 Garching, Germany*

[3] *State Key Laboratory for Artificial Microstructureand Mesoscopic Physics, School of Physics, Frontiers Science Center for Nano-optoelectronics& Collaborative Innovation Center of Quantum Matter, Peking University, Beijing, 100871, People's Republic of China*

[4] *Peking University Yangtze Delta Institute of Optoelectronics, Nantong, 226010, People's Republic of China*

5 *Collaborative Innovation Center of Extreme Optics, Shanxi University, Taiyuan, 030006, People's Republic of China*

†These authors contributed equally to this work.

*Corresponding author e-mail: sharp@wsi.tum.de (I. S.); iamzhurui@pku.edu.cn (R. Z.)

Table S1. Hall effect measurements results for ABS-OA, ABS-MPA, and ABS-MeOH.

Sample	Carrier type	Resistivity (Ω cm)	Moblity (cm^2/V s)
ABS-OA	*n*	10^5	11.5
ABS-MPA	*n*	10^3	15.5
ABS-MeOH	*p*	10^2	14.3

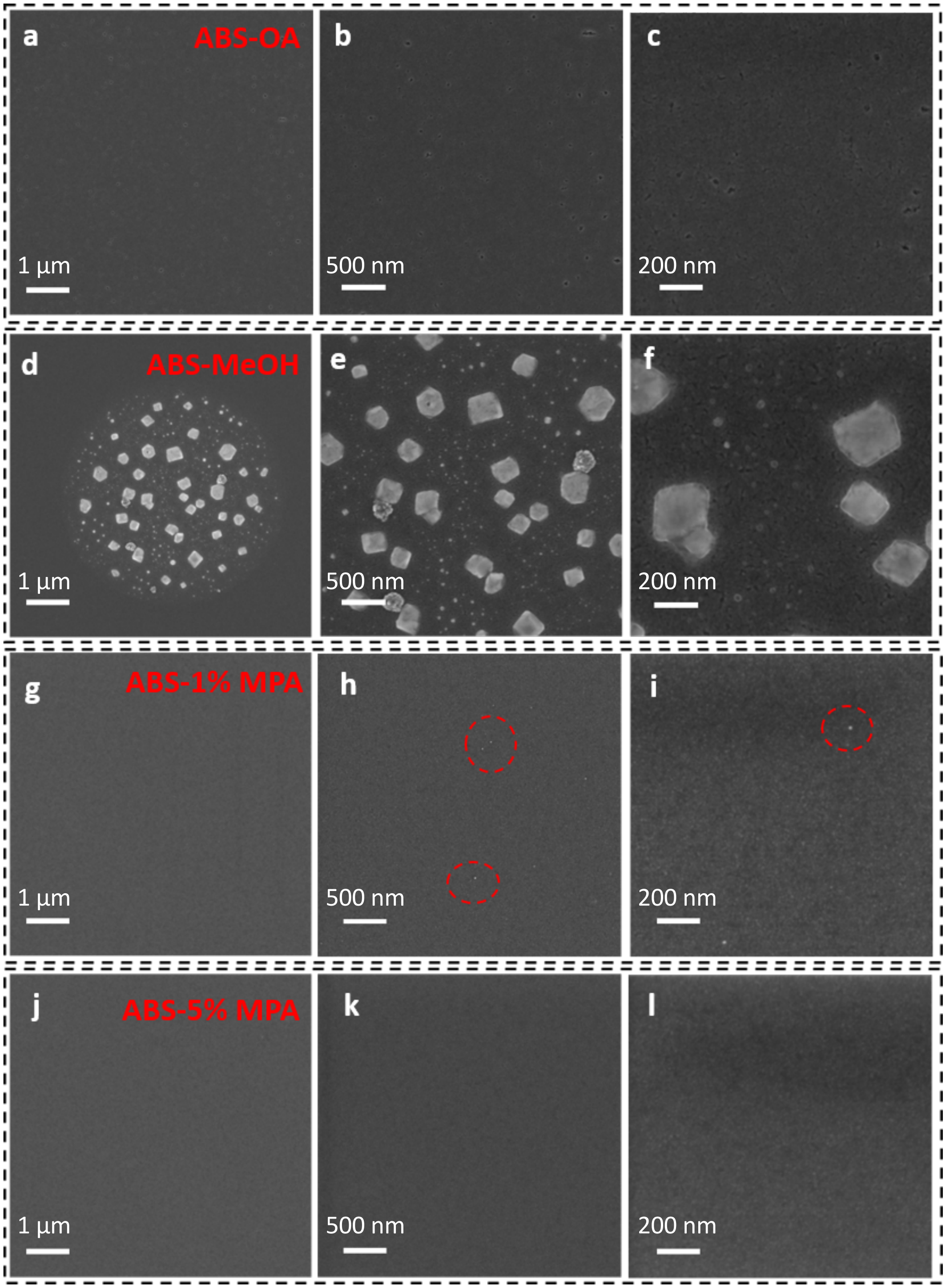

Figure S1. Scanning electron microscopy (SEM) images for (a-c) ABS-OA, (d-f) ABS-MeOH, (g-i) ABS-1 vol.% MPA, and (j-l) ABS-5 vol.% MPA on ITO substrates.

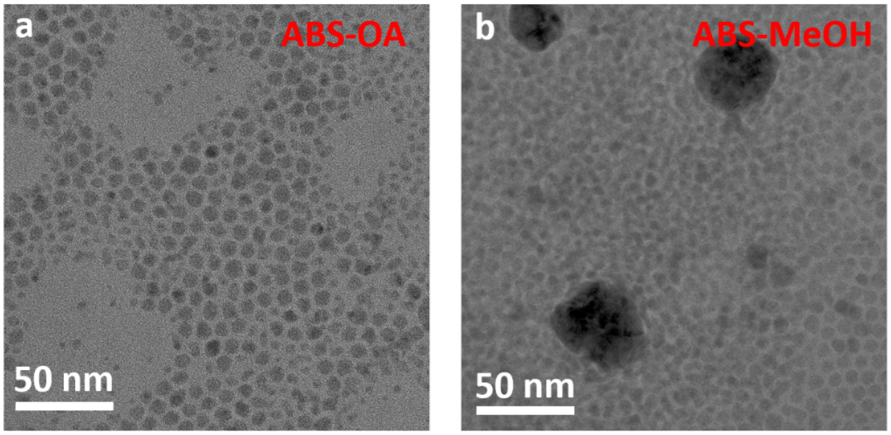

Figure S2. Transmission electron microscopy (TEM) images for (a) ABS-OA, (b) ABS-MeOH.

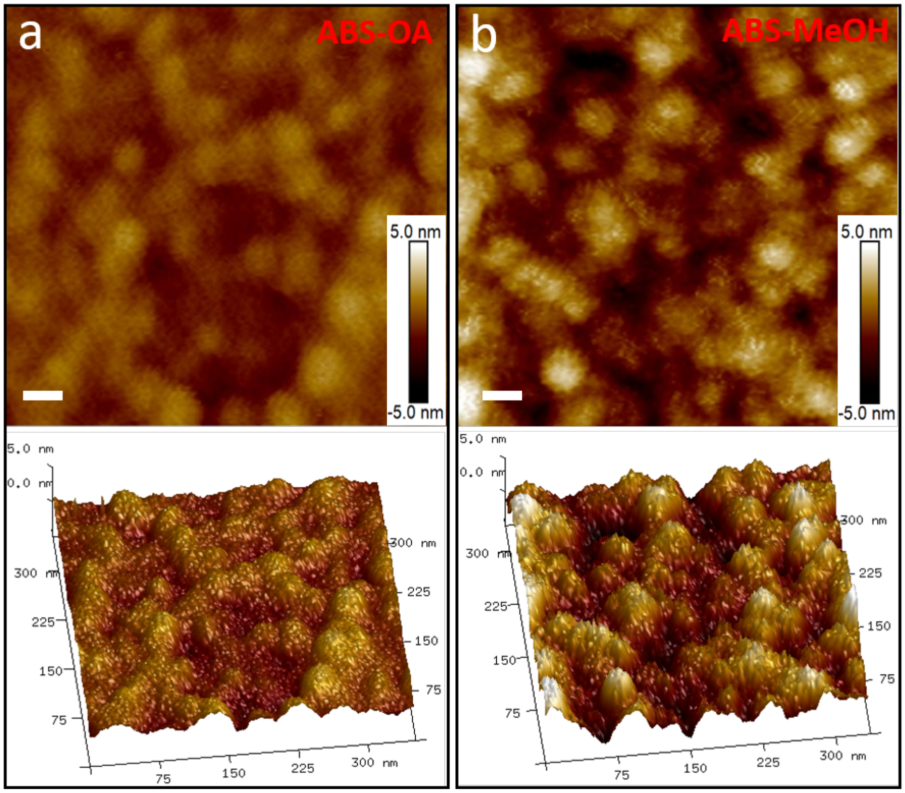

Figure S3. Atomic force microscope (AFM) images of (a) ABS-OA, (b) ABS-MeOH. Scale bar: 5 nm.

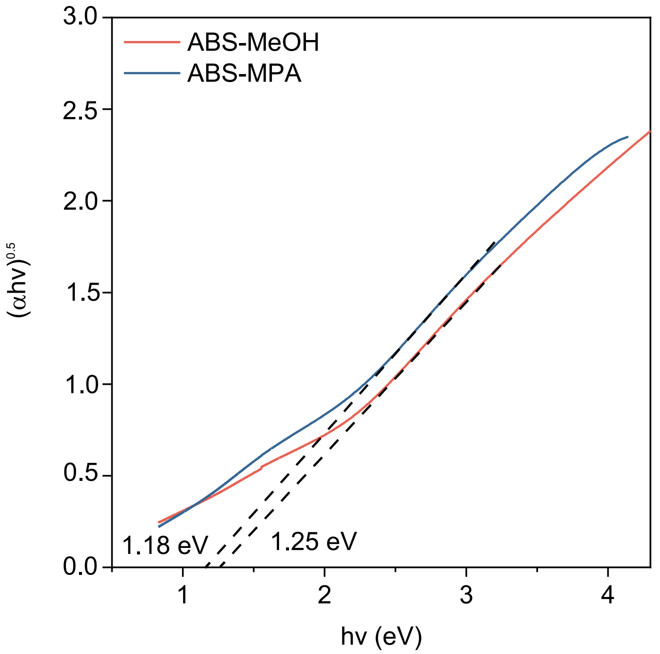

Figure S4. Tauc plots for ABS-MeOH and ABS-MPA.

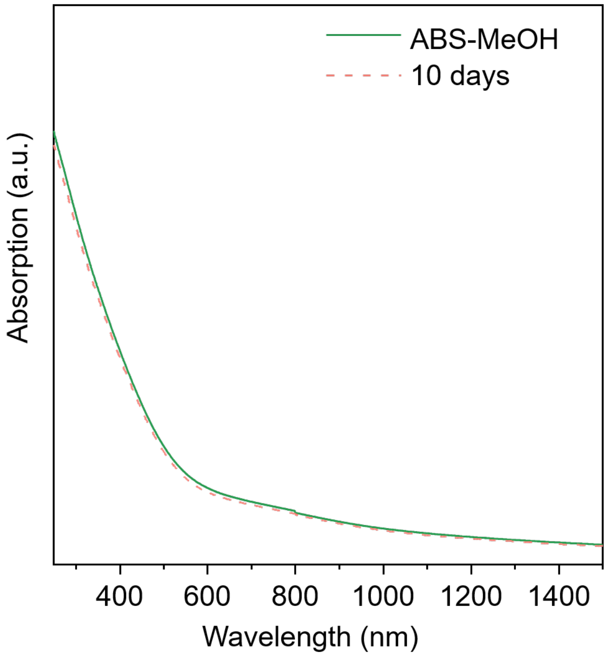

Figure S5. UV-vis absorption spectra for ABS-MeOH and ABS-MeOH stored in air after 10 days.

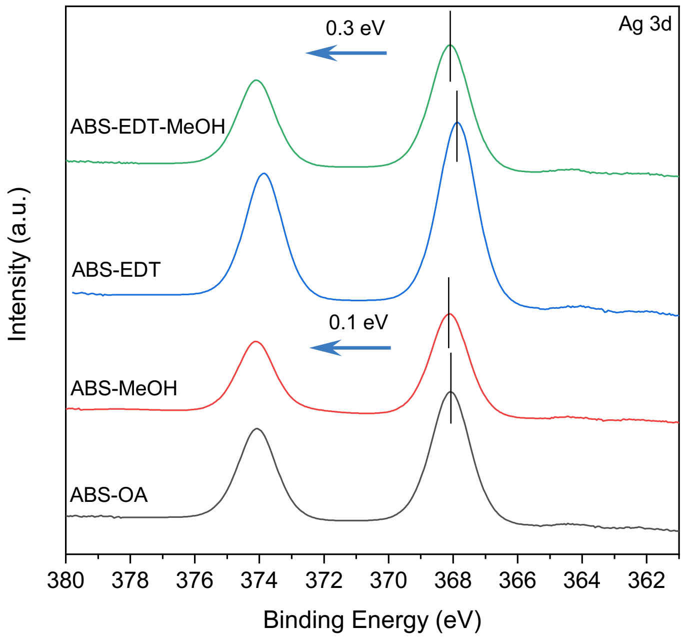

Figure S6. Ag 3*d* high-resolution XPS spectra of ABS-OA, ABS-MeOH, ABS-EDT, and ABS-EDT-MeOH.

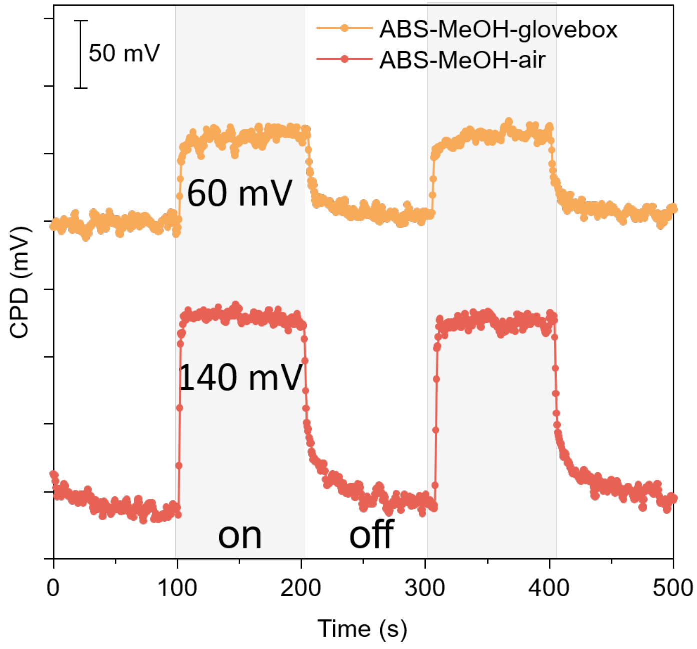

Figure S7. Change of CPD as a function of time in the dark and under 455 nm LED illumination for ABS following MeOH treatment in air and in glovebox. The grey areas indicated the times during which the samples were illuminated.

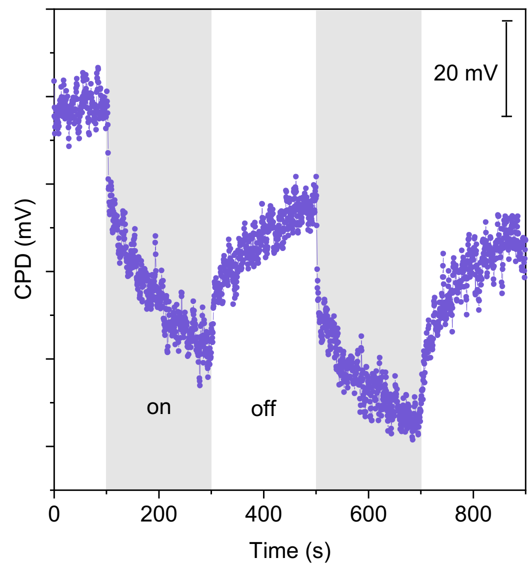

Figure S8. Change of CPD as a function of time in the dark and under 455 nm LED illumination with N_2 protection for ABS following MeOH treatment performed in an air-free glovebox. The grey areas indicated the times during which the samples were illuminated.

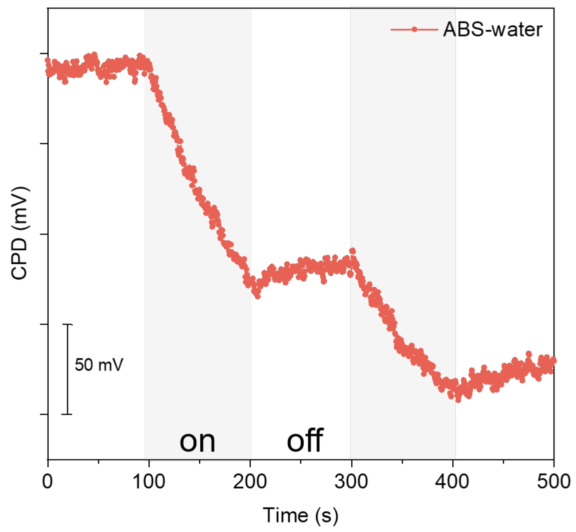

Figure S9. Change of CPD as a function of time in the dark and under 455 nm LED illumination for ABS following water treatment. The grey areas indicated the times during which the samples were illuminated.

Table S2. Dielectric constant of different solvents.

**Solvents**	Hexane	Toluene	Isopropanol	Acetone	Methanol	Acetonitrile
**Dielectric constant**	2.02	2.38	17.9	20.7	32.7	37.5

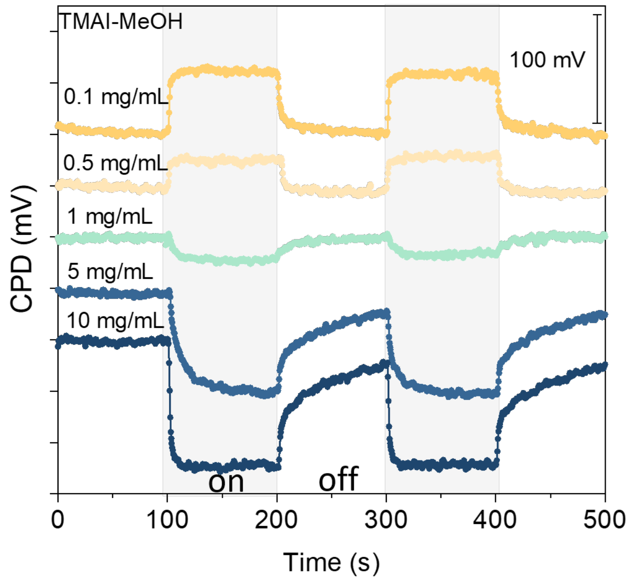

Figure S10. Change of CPD as a function of time for ABS treated by TMAI MeOH solution with varied concentration in the dark and under illumination. The grey areas indicated the times during which the samples were illuminated.

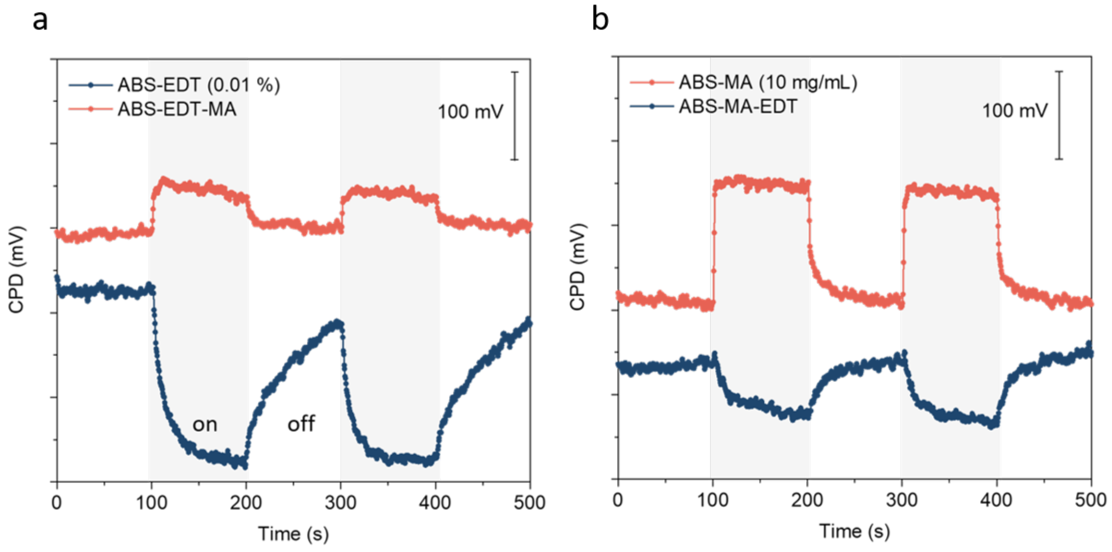

Figure S11. Change of CPD as a function of time in the dark and under 455 nm LED illumination. (a) ABS treated by 0.01 vol% EDT solution first (ABS-EDT) and ABS-EDT treated by 10 mg/mL MA solution (ABS-EDT-MA). (b) ABS treated by 10 mg/mL MA solution first (ABS-MA) and ABS-MA treated by 0.01 vol% EDT solution (ABS-MA-EDT). The grey areas indicated the times during which the samples were illuminated.

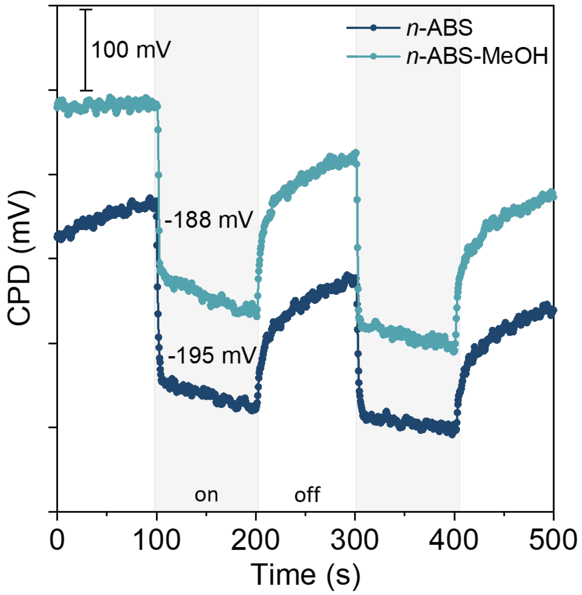

Figure S12. Change of CPD as function of time in the dark and under 455 nm LED illumination of *n*-ABS before and after MeOH treatment. The grey areas indicated the times during which the samples were illuminated.

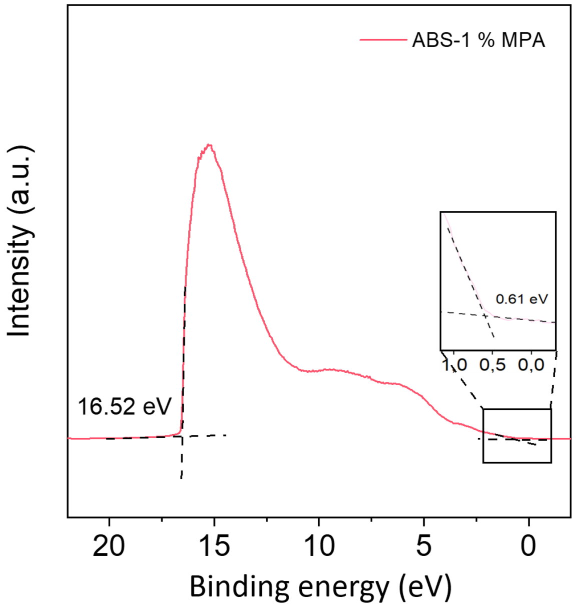

Figure S13. Ultraviolet photoelectron spectroscopy (UPS) measurements for ABS treated by 1 vol% MPA MeOH solution.

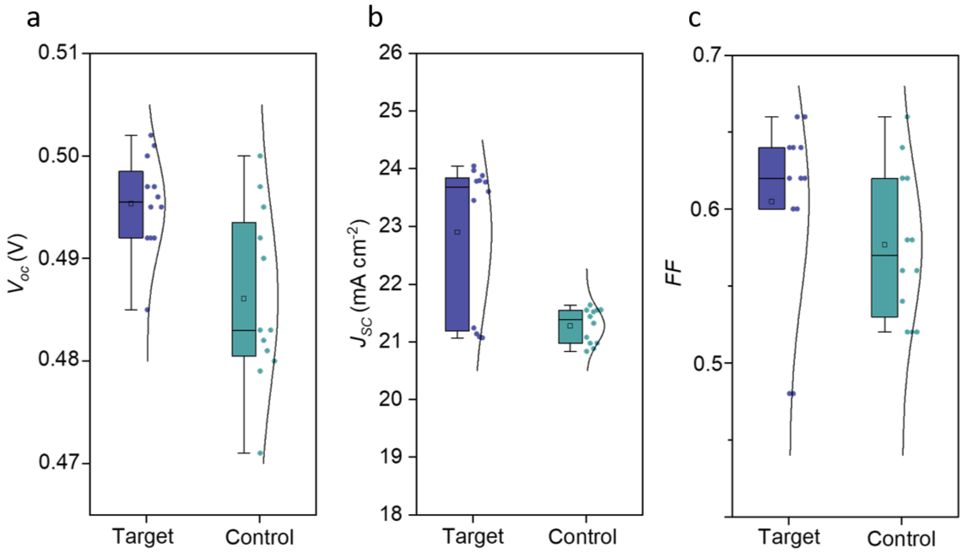

Figure S14. Statistics of target and control samples represented in box-and-whisker plots: (a) Open-circuit voltage (V_{OC}), (b) fill factor (FF), and (c) short-circuit current density (J_{SC}).

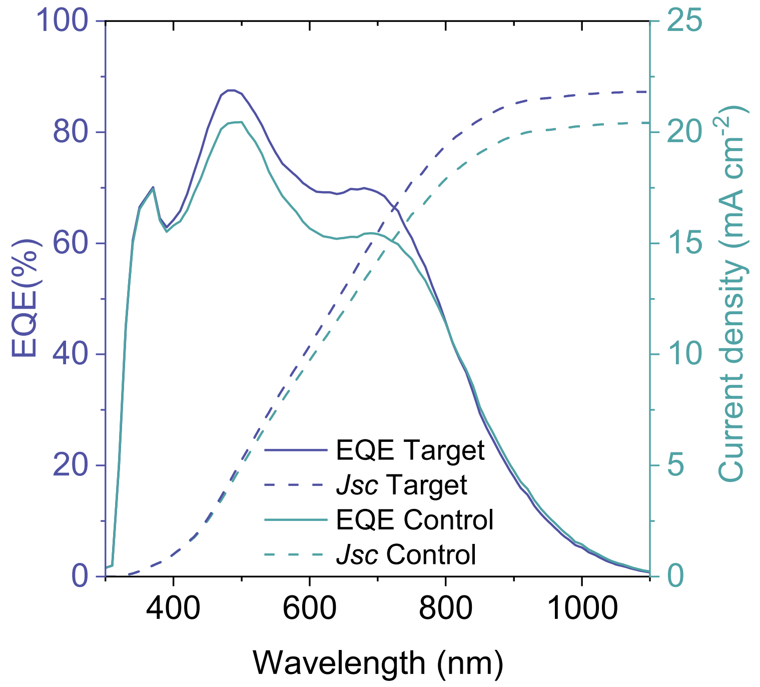

Figure S15. EQE spectra of the champion device of target sample (ABS *p-n* homojunction) and control sample (ABS-1 vol.% MPA).

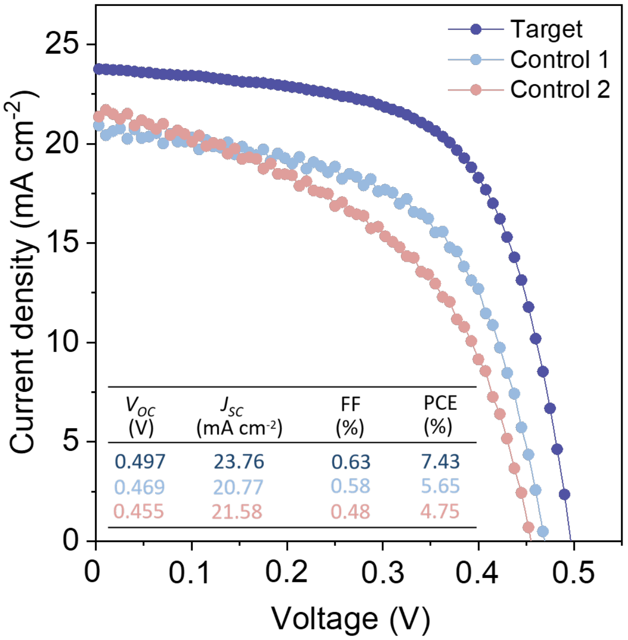

Figure S16. *J-V* curves of the champion device comprising the target sample (ABS *p-n* homojunction) compared to Control 1 sample (ITO/SnO_2/*n*-ABS/PTAA/MoO_3/Ag) and Control 2 sample (ITO/SnO_2/*p*-ABS/*n*-ABS/PTAA/MoO_3/Ag).

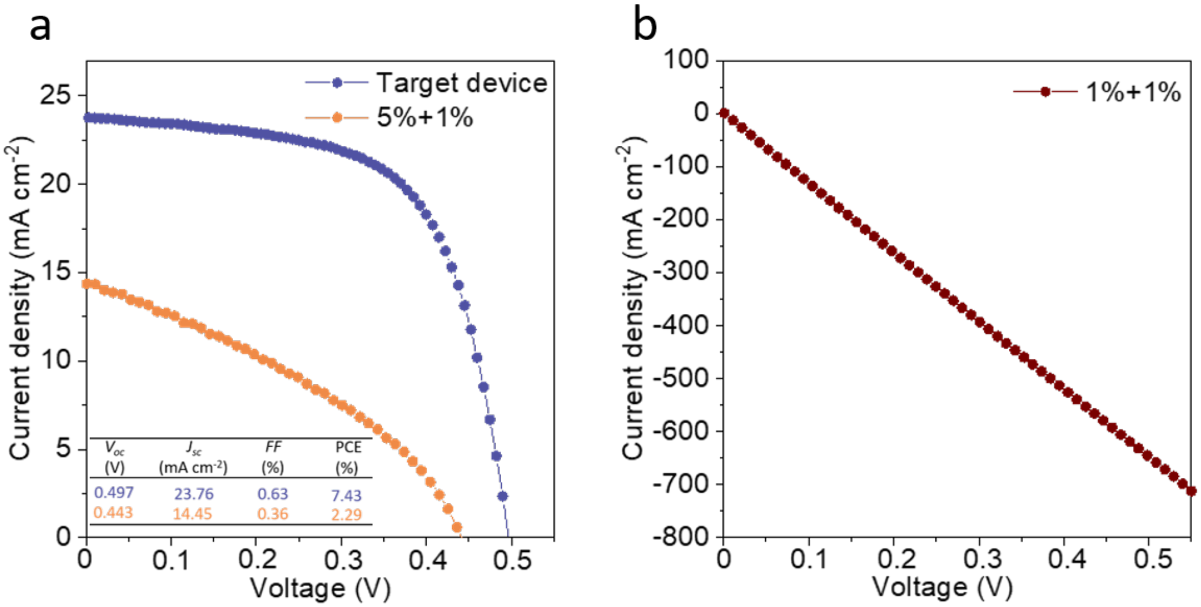

Figure S17. *J-V* curves of (a) champion device comprising the target sample (ITO/SnO_2/*n*-ABS/*p*-ABS/PTAA/MoO_3/Ag) compared to sample in absence of SnO_2 and PTAA layers (ITO/*n*-ABS/*p*-ABS /MoO_3/Ag), (b) ITO/*p*-ABS/MoO_3/Ag.

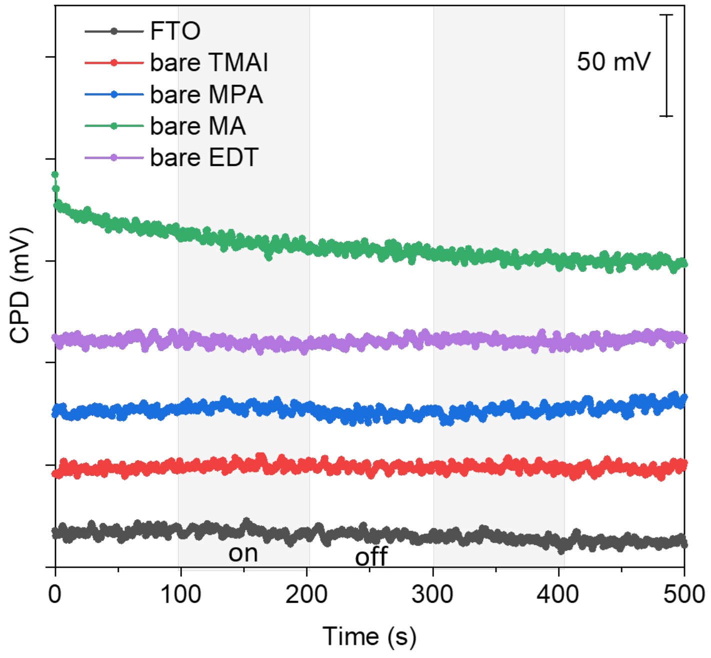

Figure S18. Change of CPD as function of time in the dark and under 455 nm LED illumination for bare FTO and different bare ligands. The grey areas indicated the times during which the samples were illuminated.

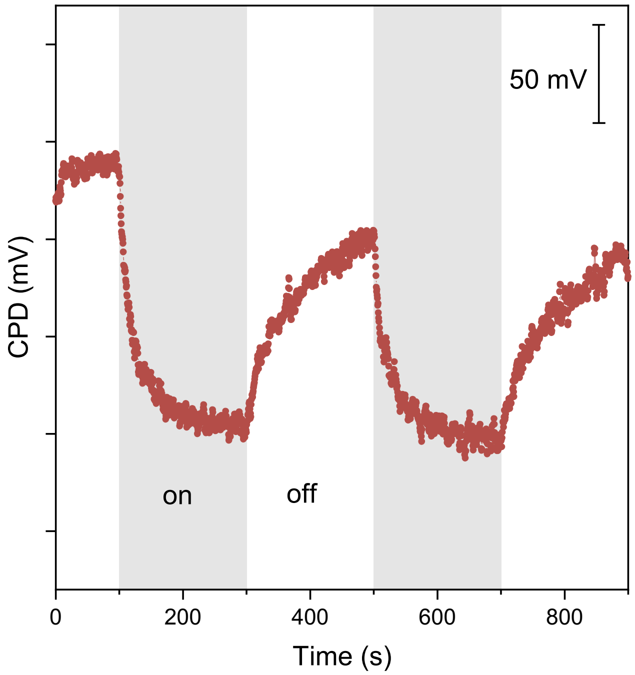

Figure S19. Change of CPD as function of time in the dark and under 455 nm LED illumination for *n*-type TiO_2.
